# Improving statin treatment strategies to reduce LDL-cholesterol: factors associated with targets’ attainment in subjects with and without type 2 diabetes

**DOI:** 10.1186/s12933-021-01338-y

**Published:** 2021-07-16

**Authors:** Mario Luca Morieri, Valentina Perrone, Chiara Veronesi, Luca Degli Esposti, Margherita Andretta, Mario Plebani, Gian Paolo Fadini, Saula Vigili de Kreutzenberg, Angelo Avogaro

**Affiliations:** 1 Department of Medicine, University of Padova, Via Giustiniani 2, 35128 Padua, Italy; 2 CliCon S.R.L. Health Economics & Outcomes Research, Bologna, Italy; 3 Assistenza Farmaceutica Territoriale, Azienda ULSS 8 Berica, Vicenza, Italy; 4 University Hospital of Padova , Padova, Italy

**Keywords:** Cardiovascular prevention, Statins, PCSK9, Ezetimibe, Gender, HDL

## Abstract

**Background:**

This cross-sectional study aimed to identify actionable factors to improve LDL-cholesterol target achievement and overcome underuse of lipid-lowering treatments in high- or very-high-cardiovascular risk patients.

**Methods:**

We evaluated healthcare records of 934,332 subjects from North-Italy, including subjects with available lipid profile and being on statin treatments up to December 2018. A 6-month-period defined adherence with proportion-of-days-covered ≥ 80%. Treatment was classified as high-intensity-statin (HIS) + ezetimibe, HIS-alone, non-HIS (NHIS) + ezetimibe or NHIS alone.

**Results:**

We included 27,374 subjects without and 10,459 with diabetes. Among these, 30% and 36% were on secondary prevention, respectively. Adherence was high (78–100%) and increased with treatment intensity and in secondary prevention. Treatment intensity increased in secondary prevention, but only 42% were on HIS. 2019-guidelines LDL-cholesterol targets were achieved in few patients and more often among those with diabetes (7.4% vs. 10.7%, p < 0.001). Patients in secondary prevention had mean LDL-cholesterol levels aligned slightly above 70 mg/dl (range between 68 and 73 mg/dl and between 73 and 85 mg/dl in patients with and without diabetes, respectively). Moreover, the differences in mean LDL-cholesterol levels observed across patients using treatments with well-stablished different LDL-lowering effect were null or much smaller than expected (HIS vs. NHIS from − 3 to − 11%, p < 0.001, HIS + ezetimibe vs. HIS—from − 4 to + 5% n.s.). These findings, given the observational design of the study, might suggest that a “treat to absolute LDL-cholesterol levels” approach (e.g., targeting LDLc of 70 mg/dl) was mainly used by physicians rather than an approach to also achieve the recommended 50% reduction in LDL-cholesterol levels. Our analyses suggested that female sex, younger age, higher HDL-c, and elevated triglycerides are those factors delaying prescription of statin treatments, both in patients with and without diabetes and in those on secondary prevention.

**Conclusions:**

Among patients on statin treatment and high adherence, only a small proportion of patients achieved LDL-cholesterol targets. Late initiation of high-intensity treatments, particularly among those with misperceived low-risk (e.g., female subjects or those with high HDL-cholesterol), appears as pivotal factors needing to be modified to improve CVD prevention.

**Supplementary Information:**

The online version contains supplementary material available at 10.1186/s12933-021-01338-y.

## Introduction

Cardiovascular disease (CVD) is the leading cause of mortality and morbidity in patients with type 2 diabetes mellitus. In these patients, dyslipidemia is a major cardiovascular risk factor. As such, the 3-hydroxy-3-methylglutaryl coenzyme A (HMG-CoA) reductase inhibitors (statins) are the best therapy for low-density lipoprotein cholesterol (LDLc) reduction. Several trials have shown significant CV risk reduction through LDLc lowering in patients with diabetes, supporting its causal role in atherogenesis, as also demonstrated in genetic studies [1]. Statin therapy is considered essential both in primary and secondary prevention of CVD in diabetes. Trials have shown that statin therapy is beneficial for people with diabetes even if they do not already have manifest coronary heart disease or high cholesterol concentrations [2]. In the Health Protection Study (HPS), allocation to 40 mg simvastatin daily reduced the rate of first major vascular events by about a quarter in the diabetic patients included in that study [3]. Key observations provide evidence that, in patients with type 2 diabetes, the benefit of statin therapy confers protection at considerably low LDLc levels of less than 1.8 mmol/l (< 70 mg/dl) [4]. Additional trials and metanalyses have highlighted a benefit by further reducing LDL cholesterol down to 55 mg/dl [5, 6]. This concept has been incorporated in most of the recent guidelines, which now recommend the initiation of statin treatment on the LDL cholesterol target levels and the CV risk profile of the patient [7]. Most of the patients referring to diabetes clinics suffer from type 2 diabetes and have an average duration of the disease > 10 years, at least 90% have high blood pressure. More than 60% are on statin treatment [8]: therefore, according to the 2019 ESC/EASD Guidelines, most of them fall into either high or very high-risk categories for which LDLc levels < 70 mg/dl and 55 mg/dl, respectively, are recommended. With the same class of recommendation and level of evidence, guidelines underscore the importance of LDLc reduction of at least 50%. These more stringent goals make the attainment of LDLc targets among diabetic patients even more problematic. In the PINNACLE registry, patients with comorbidities were less likely to have LDLc at target, particularly those with diabetes [9]. Overall, systematic reviews and national-or multinational surveys indicate that a substantial proportion of patients at high risk are exposed to an unacceptably high concentration of LDLc [10]. Previous studies have highlighted the difficulties to reach lipid targets, especially in patients with type 2 diabetes [11, 12], although a significant correlation is observed between adherence and plasma LDLc [13], the probability of goal achievement appears, and lipid management appears far from perfect [14]. Mainly routine care data have shown high off-target prevalence and little change in LDLc over time [9, 15–17]. Off-target values are persistent in diabetes mellitus, which is considered an equivalent of CV disease, also in primary prevention [11, 18].

However, comparisons between LDLc targets as a function of the type of statin treatment, adherence, prior cardiovascular event in patients with and without diabetes are lacking. Furthermore, the prevalence of off-target values, in light of the new recommended LDLc target for very high-risk patients, is unknown.

Therefore, in light of these considerations, we performed a retrospective, cross-sectional study, intending to gain a snapshot of lipid levels of two different populations, with and without diabetes, in the light of a previous cardiovascular event. More specifically, we wished to: (1) determine the proportion of patients, on different types of statin treatments, at target for LDLc; (2) analyze the differences between patients without and with diabetes; (3) quantify the impact of prior events; (4) identify the determinants of LDLc attainment and quantify their contribution.

## Methods

### Data source

To accomplish our aims, we have obtained: (1) Administrative data of the Padova Regional Health System District; (2) Central Laboratory data from the University Hospital of Padova using anonymized claims data. For the administrative data, we have integrated four primary sources: (a) subject-specific and co-payment exemption data from the regional registry of healthcare beneficiaries, which records each citizen's reference local healthcare unit, start and end of the residency in the Veneto Region, and their exemption from co-payment (due to illness or income); (b) prescription medicine data including the Anatomical-Therapeutic Chemical (ATC) code and detailed pharmacy-claim with information on the dose of the package dispensed to the patients; (c) hospitalization-related diagnoses at hospital discharge, and procedures during the hospital stay, recorded using ICD-9-CM codes; (d) visits in outpatient clinics at a university-affiliated tertiary care center [19].

For the present analysis, all the data used were previously anonymized as per the law concerning their research and governance purposes. As requested by the local Institutional Review Board, the researchers only accessed a non-identifiable dataset.

### Study population

The enrolment period started on the first of January 2014 and ended 31 December 2018, which corresponds to the end of data availability. All the subjects included in the present analysis had at least one routine laboratory lipid analysis report: total cholesterol, LDLc, and HDL cholesterol, between 1/01/2014 and 31/12/2018 from the Laboratory of the University Hospital of Padova and a concomitant statin treatment (Atorvastatin—ATC code C10AA01, Lovastatin—ATC code C10AA02, Pravastatin—ATC code C10AA03, Fluvastatin—ATC code C10AA04, Atorvastatin—ATC code C10AA05, Rosuvastatin—ATC code C10AA07, Simvastatin + Ezetimibe—ATC code C10BA02, Atovarvastatin + Ezetimibe—ATC code C10BA05, Rosuvastatin + Ezetimibe—ATC code C10BA06).The index date for each patient included in the study was defined as the last LDLc determination date within the enrolment period. The index date can occur during the enrolment period and marked the moment from which no other LDLc determination was observed until the end of data availability.

Additional clinical data available for the present analysis were: triglycerides, creatinine, and glycated hemoglobin (HbA1c). The patients included in the current analysis had at least one determination of these parameters within the year before the index date. Non-HDL cholesterol is defined as the total cholesterol value minus HDL cholesterol value. The estimated glomerular filtration rate was calculated according to the Chronic Kidney Disease Epidemiology Collaboration (CKD-EPI).

Patients had the diagnosis of diabetes and were identified as such if they had a hospital diagnosis code ICD-9-CM 250 or at least two prescriptions of antidiabetic medication (ATC code A10) in the 12 months before the index date. Exclusion criteria were all patients treated with monoclonal antibodies against Proprotein convertase subtilisin/Kexin type 9 (PCSK9i) (ATC codes C10AX13, C10AX14) before the index date.

### CV event assessment

As shown in Additional file 1: Figure S1A, cardiovascular events were identified starting from 10/01/2010 based on the first six diagnosis codes and admission time reported in the hospital discharge claims database, that is, by mapping ICD-9-CM codes to the appropriate outcomes: diagnosis codes 410–414 to infarction, 430–438 to stroke, 390–459 to hospitalization for cardiovascular causes; procedure codes 00.55, 00.61–00.66, 36.03, 36.06–36.07, 36.1, 38.48, 39.50, 39.52, 39.71, and 39.90 to revascularization. Patients were defined as having cerebrovascular disease before the index date if the diagnosis code, either principal or secondary, was identified with the following ICD-9 codes: 430, 431, 432, 433, 434, 435, 436, 437, 438. Patients were defined as having coronary heart disease (CHD) before the index date if the diagnosis code, either principal or secondary, was identified with the following ICD-9-CM codes: 414 or procedure codes 00.55, 00.61–00.66, 36.03, 36.06–36.07, 36.1. Patients were defined as having coronary heart disease before the index date if the diagnosis code, either principal or secondary, was identified with the following ICD-9 codes: 443.9. We have classified as hypertensive patients who had in the year before the index date at least two prescriptions of antihypertensive medication (ATC codes: C03, C07, C08, C09). In the present analysis, the following statin treatments have been categorized into high-intensity statin (HIS) treatments: Atorvastatin 40 mg and 80 mg, Rosuvastatin 20 mg, and 40 mg, Atorvastatin + Ezetimibe 40/10 and 80/10 mg, Rosuvastatin + Ezetimibe 20/10 and 40/10 mg. Other statin treatments have been categorized as non-high-intensity statin (NHIS) treatments. We stratified patients in the following group of increasing LDL-c lowering effect: NHIS, NHIS + ezetimibe, HIS, HIS + ezetimibe.

### Adherence assessment

Adherence to statin treatment has been estimated 6 months before the index date using the proportion of days covered (PDC) method [20], i.e., the ratio between the number of days of medication supplied (according to pharmacy-claim) and days of observation before the index date, multiplied by 100. Patients were considered adherent to statin treatment if they had a PDC of ≥ 80%.

### Risk level identification and LDLc targets

We have categorized patients with or without diabetes according to the presence/absence of previous cardiovascular events. The current LDLc target in patients with very high cardiovascular risk (including those with or without diabetes with prior history of the cardiovascular event) requires both the following criteria: more than 50% reduction from baseline LDLc levels and achievement of absolute LDLc levels < 55 mg/dl. We also evaluated the less stringent goal of absolute LDLc < 70 mg/dl (OR 50% reduction from baseline) since these were recommended by the guidelines available at the time when these data were collected (i.e., according to EAS/ESC 2016 Guidelines) [21]. According to EAS/ESC guidelines, we considered only HIS and HIS + ezetimibe as treatments allowing to reduce LDLc levels of at least 50% from pre-treatment levels [21, 22].

### Statistical analysis

Continuous data are presented as means ± standard deviation and categorical variables as numbers and percentages. The analysis of variance (ANOVA) test and the chi-square test were used to compare continuous variables and categorical variables, respectively. The relationship between diabetes and the probability of achieving LDLc targets among secondary prevention subjects was reported as relative risk (relative risk with 95% CI). Logistic models were performed to analyze predictors of the LDL cholesterol < 70 mg/dl in patients with and without diabetes. Models were adjusted for prespecified covariates: age, gender, HDLc, triglycerides, estimated glomerular filtration rate (eGFR), presence of previous cardiovascular events, type of statin treatment, and adherence to statin therapy. These analyses were conducted on a complete-case dataset. Effect sizes were reported as odds ratios (OR) and 95% confidence intervals (CI). The results were considered statistically significant when the p-value was < 0.05. All statistical analyses were performed using STATA SE software version 12.1 (StataCorp LP, College Station, Texas).

## Results

### Main characteristics, statin/ezetimibe treatment, and adherence.

Among the 934,332 citizens assisted by the Euganea Veneto Regional Health Service, 241,712 had at least one lipid determination between January 1st, 2014 and December 31st, 2018 (Additional file 1: Figure S1B), and 37,883 (16%) of these were on statin therapy (72% without diabetes and 28% with diabetes). The prevalence of cardiovascular events was higher among patients with diabetes than that without diabetes (36% vs. 30% p < 0.0001).

Overall, as shown in Table 1, most of the patients were treated with NHIS (81%) and only 19% with HIS, and 9% of subjects were on concomitant treatment with ezetimibe (7% on NHIS + ezetimibe and 2% on HIS + ezetimibe). The proportion of women on statin treatment was overall 47%, and it was progressively reduced with the increasing intensity of lipid-lowering treatments (from 51% on NHIS to 30% in HIS + ezetimibe, p < 0.001 for trend; all between-groups comparison were statistically significant after Bonferroni correction, except HIS vs HIS + ezetimibe). The prevalence of diabetes was similar across different treatment groups, while as expected, prevalence of CVD was progressively higher with increasing intensity of treatments. The eGFR in patients with or without diabetes was lower in those with a prior event (Tables 2 and 3). Patients with diabetes were on reasonably good metabolic control as testified by HbA1c levels, and a significant proportion of them was on insulin treatment.

**Table 1:** General characteristics of the population included in the study

	NHIS	NHIS + Eze	HIS	HIS + Eze	p value*
	N = 27,795 (74%)	N = 2815 (7%)	N = 6578 (17%)	N = 645 (2%)	
Age in years mean (S.D.)	72.6 (11)	69.4 (11)	71.7 (11)	66.9 (11)	< 0.001
Female n (%)	14,145 (51)	1202 (43)	2157 (33)	196 (30)	< 0.001
Age < 80 years n (%)	20,695 (75)	2411 (86)	5026 (76)	597 (93)	< 0.001
Hypertension n (%)	21,712 (78)	2296 (82)	5789 (88)	579 (90)	< 0.001
Diabetes n (%)	7718 (28)	772 (27)	1821 (28)	148 (23)	N.S
GFR (ml/min/1.73 m^2^) mean (S.D.)	89.9 (31)	85.1 (30)	89.4 (32)	91.9 (31)	< 0.001
HbA1c (mmol/mol) mean (S.D.)	47.6 (13)	46.6 (12)	46.2 (13)	45.0 (12)	< 0.001
CVD n (%)	2592 (9)	573 (20)	1816 (28)	287 (45)	< 0.001
Cerebrovascular disease n (%)	2355 (9)	257 (9)	1838 (28)	72 (11)	< 0.001
Peripheral vascular disease n (%)	12 (0)	N.I	N.I	0 (0)	N.S
Total-chol (mg/ml) mean (S.D.)^a^	167.8 (39)	162.4 (42)	145.1 (39)	145.3 (41)	< 0.001
LDL-chol (mg/dl) mean (S.D.)^a^	94.1 (32)	88.1 (35)	78.8 (31)	79.6 (35)	< 0.001
HDL-chol (mg/dl) mean (S.D.)^a^	52.8 (16)	51.8 (15)	46.8 (15)	47.3 (13)	< 0.001
Triglycerides (mg/dl) mean (S.D.)^a^	98.7 (62)	103.9 (69)	89.9 (64)	82.1 (52)	< 0.001
Non-HDL-chol (mg/dl) mean (S.D.)^a^	115.0 (35)	110.6 (39)	98.3 (35)	98 (39)	< 0.001
ACEi/ARB n (%)	17,097 (62)	1793 (64)	4570 (70)	472 (73)	< 0.001
Beta blockers n (%)	8923 (32)	1223 (43)	3482 (53)	435 (67)	< 0.001
CCB n (%)	6690 (24)	677 (24)	1716 (26)	138 (21)	< 0.01
Treat. Diabetes n (%)	7373 (27)	728 (26)	1665 (25)	134 (21)	< 0.01
Fibrates n (%)	185 (0.7)	31 (1.1)	50 (0.8)	5 (0.8)	N.S
Simvastatin n (%)	10,350 (37.2)	44 (1.6)	0 (0.0)	0 (0.0)	
Lovastatin n (%)	840 (3.0)	30 (1.1)	0 (0.0)	0 (0.0)	
Pravastatin n (%)	1297 (4.7)	44 (1.6)	0 (0.0)	0 (0.0)	
Fluvastatin n (%)	256 (0.9)	5 (0.2)	0 (0.0)	0 (0.0)	
Atorvastatin n (%)	12,003 (43.2)	205 (7.3)	5864 (89.0)	516 (80.0)	
Rosuvastatin n (%)	3049 (11.0)	116 (4.1)	712 (11.0)	113 (17.5)	
Simvastatin + Ezetimibe n (%)	0 (0.0)	2363 (83.9)	0 (0.0)	0 (0.0)	
Rosuvastatin + Ezetimibe n (%)	0 (0.0)	8 (0.3)	N.I	16 (2.5)	

Data reported as mean (S.D.) or as n (%). Opinion 05/2014 on “Anonymisation Techniques” drafted by the “European Commission Article 29 Working Party”, the analyses involving less than three patients were not reported, as potentially reconductable to single individuals. Therefore, results referred to ≤ 3 patients were reported as NI (not issuable)

*HIS* high-intensity statins, *NHIS* non-HIS, *Eze* ezetimibe

^*^ANOVA P value for differences between groups (i.e., if < 0.05 not all group are equivalent)

^a^Analyses performed on patients with at least one detection before index

**Table 2:** Characteristics of patients without diabetes according to concomitant events.

	1. No-diabetes w/o event					2. No-diabetes with event				
	NHIS	NHIS + Eze	HIS	HIS + Eze	p value*	NHIS	NHIS + Eze	HIS	HIS + Eze	p value*
Nr. patients	16,131 (85)	1305 (7)	1492 (8)	123 (1)		3946 (47)	738 (9)	3265 (39)	374 (5)	
Adherent	12,586 (78)	1091 (84)	1213 (81)	112 (91)	< 0.001	3186 (81)	660 (89)	2767 (85)	356 (95)	< 0.001
Total-chol (mg/dl)^a^	180.3 (37)	178.5 (43)	168.7 (43)	170.1 (46)	< 0.001	152.6 (39)	150.2 (36)	139.1 (33)	136.6 (33)	< 0.001
LDL-chol (mg/dl)^a^	103.8 (31)	100.8 (36)	95.6 (36)	100 (42)	< 0.001	84.5 (31)	79.5 (28)	75.4 (27)	72.7 (26)	< 0.001
HDL-chol (mg/dl)^a^	56.2 (16)	55.6 (15)	52.7 (16)	52.5 (14)	< 0.001	49 (16)	50.6 (15)	46.5 (14)	47.2 (13)	< 0.001
Triglycerides (mg/dl)^a^	97.6 (58)	102.9 (65)	97.2 (66)	85.1 (47)	0.001	86.4 (59)	92.4 (65)	78.7 (54)	74 (48)	< 0.001
NON-HDL-chol (mg/dl)^a^	124.1 (34)	122.8 (42)	116 (40)	117.6 (45)	< 0.001	103.6 (35)	99.6 (33)	92.6 (30)	89.4 (31)	< 0.001
GFR (ml/min/1.73 m^2^)^a^	91.8 (26)	89.6 (25)	95.2 (31)	93.4 (44)	< 0.001	80.4 (34)	80.3 (30)	89.6 (30)	92.3 (23)	< 0.001
HbA1c (mmol/mol)^a^	41 (8)	40.8 (6)	41.3 (7)	40.7 (6)	N.S	40 (7)	39.7 (5)	39.6 (6)	40.6 (8)	N.S
CHD						1672 (42)	398 (54)	1197 (37)	215 (58)	< 0.001
Cerebrovascular disease						1565 (40)	171 (23)	1307 (40)	44 (12)	< 0.001
PAD						4 (0)	N.I	N.I	0 (0)	N.S
ACEi/ARB	9001 (56)	710 (54)	906 (61)	77 (63)	0.001	2608 (66)	520 (71)	2269 (70)	278 (74)	< 0.001
Beta blockers	4178 (26)	359 (28)	539 (36)	51 (42)	< 0.001	2041 (52)	491 (67)	1914 (59)	277 (74)	< 0.001
CCB	3183 (20)	248 (19)	296 (20)	16 (13)	N.S	1096 (28)	183 (25)	782 (24)	72 (19)	< 0.001
Fibrates	83 (1)	7 (1)	12 (1)	N.I	N.S	16 (0)	7 (1)	9 (0)	N.I	N.S

Opinion 05/2014 on “Anonymisation Techniques” drafted by the “European Commission Article 29 Working Party”, the analyses involving less than three patients were not reported, as potentially reconductable to single individuals. Therefore, results referred to ≤ 3 patients were reported as NI (not issuable)

*CHD* coronary heart disease, *PAD* peripheral artery disease, *ACEi* angiotensin converting-enzyme inhibitors, *ARBs* angiotensin II receptor blocker, *CCB* calcium channel blockers

^*^ANOVA P value for differences between groups (i.e., if < 0.05 not all group are equivalent)

^a^Analyses performed on patients with at least one detection before index

**Table 3:** Characteristics of patients with diabetes according to concomitant events

	3. Diabetes w/o event					4. Diabetes with event				
	NHIS	NHIS + Eze	HIS	HIS + Eze	p value*	NHIS	NHIS + Eze	HIS	HIS + Eze	p value*
Nr. patients n (%)	5661 (85)	447 (7)	532 (8)	33 (1)		2057 (54)	325 (9)	1289 (34)	115 (3)	
Adherent n (%)	4777 (84)	404 (90)	461 (87)	33 (100)	< 0.001	1677 (82)	294 (91)	1085 (84)	106 (92)	< 0.001
Total-chol (mg/dl)^a^	154.2 (34)	153.7 (37)	149.8 (37)	155.3 (48)	N.S	138.8 (37)	136.9 (34)	130.5 (35)	136.6 (44)	< 0.001
LDL-chol (mg/dl)^a^	81.7 (27)	79.6 (30)	79.2 (29)	87.5 (39)	N.S	73.1 (28)	68.8 (27)	67.6 (27)	72.1 (36)	< 0.001
HDL-chol (mg/dl)^a^	49.5 (15)	48.7 (14)	46.4 (14)	43 (9)	< 0.001	42.6 (14)	43.7 (14)	41.1 (13)	41.5 (12)	< 0.01
Triglycerides (mg/dl)^a^	108.9 (70)	122.5 (79)	114.3 (74)	109.2 (65)	< 0.001	100.7 (68)	106.6 (71)	97.6 (71)	96 (61)	N.S
NON-HDL-chol (mg/dl)^a^	104.7 (32)	105 (35)	103.4 (34)	112.3 (48)	N.S	96.2 (34)	93.2 (31)	89.4 (33)	95.1 (43)	< 0.001
GFR (ml/min/1.73 m^2^)^a^	94.7 (33)	92.5 (31)	93.8 (32)	98.7 (29)	N.S	75.2 (37)	71.8 (37)	82 (36)	86.5 (40)	< 0.001
HbA1c (mmol/mol)^a^	55.7 (13)	56.4 (13)	55.8 (13)	56 (15)	N.S	55.1 (14)	54.4 (14)	56.2 (15)	56.9 (14)	N.S
CHD						920 (45)	175 (54)	619 (48)	72 (63)	< 0.001
Cerebrovascular disease						790 (38)	86 (27)	531 (41)	28 (24)	< 0.001
PAD						8 (0)	N.I	N.I	0 (0)	N.S
ACEi/ARB	3986 (70)	320 (72)	395 (74)	26 (79)	N.S	1502 (73)	243 (75)	1000 (78)	91 (79)	< 0.05
Beta blockers	1588 (28)	152 (34)	213 (40)	16 (49)	< 0.001	1116 (54)	221 (68)	816 (63)	91 (79)	< 0.001
CCB	1669 (30)	127 (28)	147 (28)	7 (21)	N.S	742 (36)	119 (37)	491 (38)	43 (37)	N.S
Fibrates	64 (1)	12 (3)	14 (3)	0 (0)	< 0.01	22 (1)	5 (2)	15 (1)	N.I	N.S
Insulin	1366 (24)	136 (30)	154 (29)	9 (27)	< 0.01	823 (40)	144 (44)	498 (39)	49 (43)	N.S
Metformin	3724 (66)	258 (58)	333 (63)	25 (76)	< 0.01	906 (44)	132 (41)	643 (50)	59 (51)	= 0.001
Sulfonylureas	1131 (20)	88 (20)	100 (19)	N.I	N.S	290 (14)	39 (12)	174 (14)	11 (10)	N.S
DPP-4	300 (5)	36 (8)	25 (5)	N.I	N.S	171 (8)	29 (9)	89 (7)	7 (6)	N.S
GLP-1	160 (3)	17 (4)	17 (3)	N.I	N.S	29 (1)	8 (3)	26 (2)	5 (4)	N.S
SLGT2	69 (1)	12 (3)	11 (2)	0 (0)	< 0.05	24 (1)	6 (2)	17 (1)	6 (5)	< 0.01

Opinion 05/2014 on “Anonymisation Techniques” drafted by the “European Commission Article 29 Working Party”, the analyses involving less than three patients were not reported, as potentially reconductable to single individuals. Therefore, results referred to ≤ 3 patients were reported as NI (not issuable)

*CHD* coronary heart disease, *PAD* peripheral artery disease, *ACEi* angiotensin converting-enzyme inhibitors, *ARBs* angiotensin II receptor blocker, *CCB* calcium channel blockers

^*^ANOVA P value for differences between groups (i.e., if < 0.05 not all group are equivalent)

^a^Analyses performed on patients with at least one detection before index

**Fig. 1:**
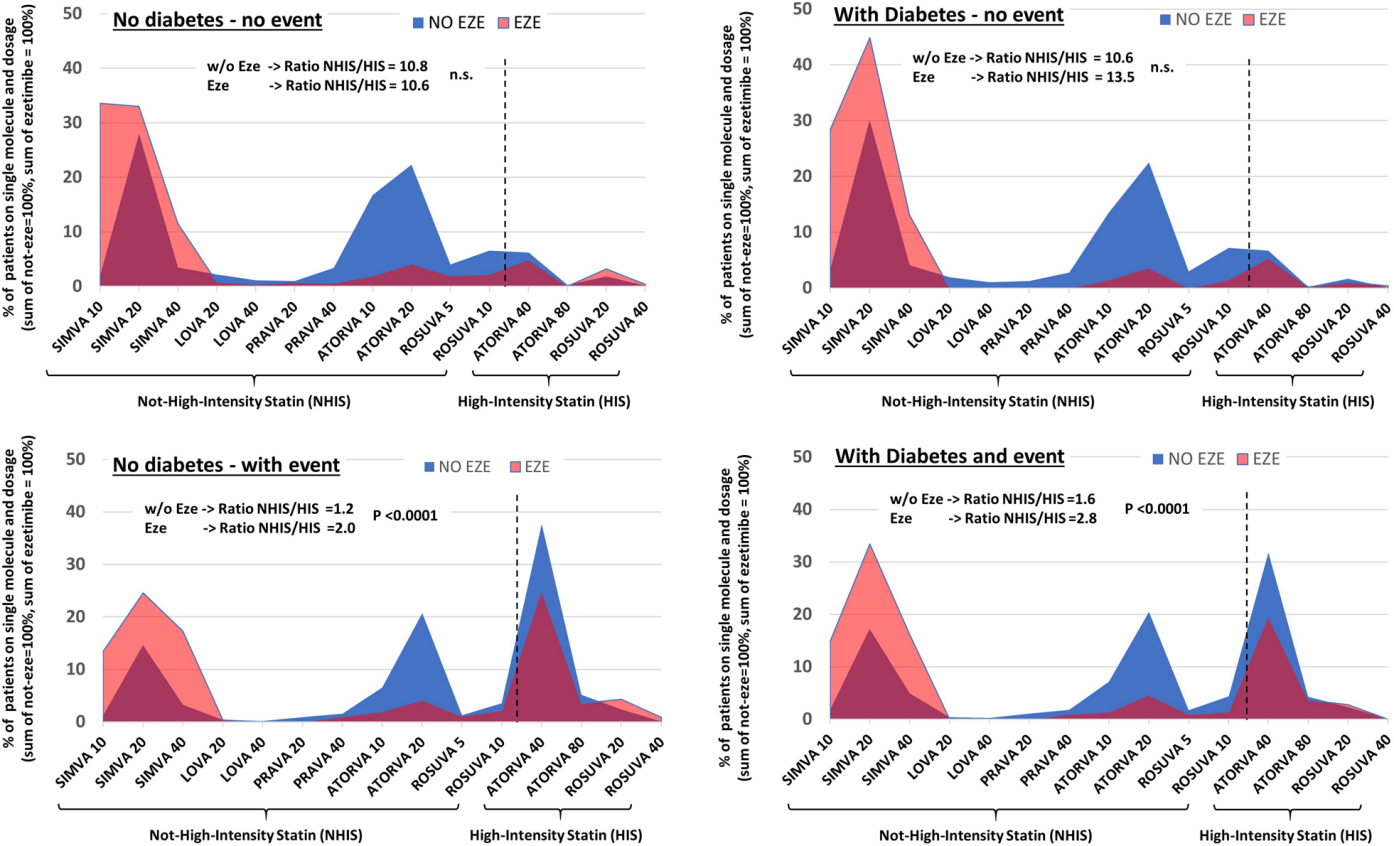
Proportion of patients (in %) on each single molecule and dosage according to presence or absence of diabetes and recorded cardiovascular events. Area under the blue area (i.e. statin alone) is 100%, sum of pink area (i.e., statin + ezetimibe) is 100%, in each of the four panel

As reported in Tables 2 and 3, treatment with HIS (either alone or with Ezetimibe) was fourfold higher in those with an event compared to those without an event, both in patients with and without diabetes (8.5% vs. 37.0% and 8.4% vs. 43.7%, respectively). However, the addition of Ezetimibe among patients with previous events (as graphically depicted in Fig. 1) was mostly prescribed in combination with NHIS and only in a few cases with HIS (NHIS/HIS ratio significantly greater in the ezetimibe group). Conversely, among subjects in primary prevention, NHIS/HIS ratio was similar between subjects with/without Ezetimibe, but Ezetimibe was mainly used in combination with low dose statin (i.e., simvastatin 10 or 20 mg in more the two-third of patients). In both populations, with and without diabetes, the treatment adherence was significantly higher in those with a previous event and increased with increasing treatment intensity [being higher among those subjects on high-intensity statins + Ezetimibe, as reported in Tables 2 and 3, were, beyond the significant ANOVA test, between-group comparison showed that the adherence was consistently significantly higher in patients treated with HIS + ezetimbe as compared to those on NHIS (all p < 0.05 after Bonferroni correction)].

### LDL and non-HDL levels across different treatments

As reported in Tables 2 and 3, patients with prior events, either with or without diabetes, had lower LDLc and non-HDLc concentrations across a different combination of treatments. Moreover, irrespective of the type of therapy and previous cardiovascular events, the LDLc and Non-HDLc concentrations were significantly lower in patients with diabetes (all p for comparison between groups < 0.01), except for those with the previous event treated with HIS + ezetimibe where LDLc were similar (p = 0.8).

Notably, both in subjects with and without diabetes, when we compared those treated with HIS with those treated with NHIS, we found, on average, only minor differences in LDLc concentration (w/o diabetes: − 8% to − 11%; diabetes: − 3% to − 8%, all p < 0.05). These were indeed markedly lower than the 28% reduction expected on average when shifting from NHIS to HIS (as reported by dyslipidemia guidelines) [22]. Even more strikingly, we found no significant differences (with the paradoxical trend towards higher levels) between-subjects on HIS + ezetimibe vs. HIS (w/o diabetes: − 4 to + 5%; diabetes + 7% to + 10% n.s.), as compared to the 30% reduction expected from RCTs and reported in guidelines [22].

### LDLc and non-HDLc levels according to the event site

Similar results were found when the population was stratified according to the event site (Additional file 1: Figure S2): in those with a previous CHD event, both the LDLc and the non-HDLc concentrations were significantly lower in patients with diabetes (71 ± 26 vs. 82 ± 29 mg/dl, p < 0.0001 for NHIS, 65 ± 24 vs. 79 ± 29 for NHIS + ezetimibe, 68 ± 27 vs. 77 ± 25 for HIS) than in those without diabetes except for the HIS + ezetimibe group. Similar results were seen for the non-HDLc. While in patients without diabetes, there were consistently lower concentrations according to statin intensity, this was not observed in patients with diabetes. In patients with cerebrovascular disease, significantly lower concentrations were observed in patients with diabetes only in those on NHIS and HIS treatment. Also, in these patients, a gradient toward significantly lower levels according to statin intensity was observed only in patients without diabetes.

### Patients at target

**Fig. 2:**
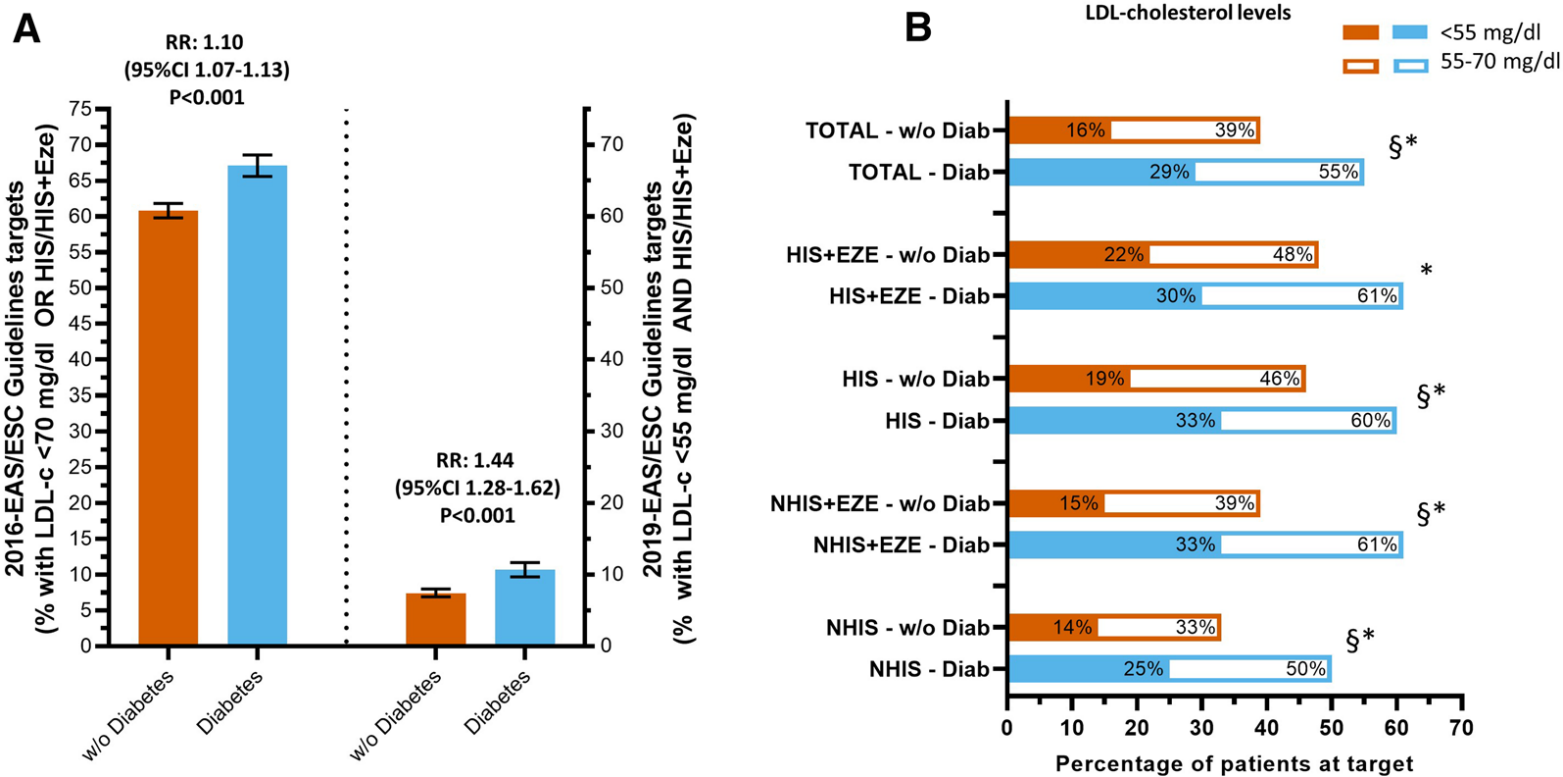
Patients with event achieving guidelines recommended targets. Error Bar represents 95% CI; § for p < 0.001 Diabetes vs. w/o Diabetes at < 55 mg/dl and * for p < 0.001 Diabetes vs. w/o Diabetes at < 70 mg/dl

As shown in Fig. 2A, among subject with prior events, only two-thirds of subjects achieved the 2016-recommended targets. This proportion dropped to one out of ten subjects when the 2019-guidelines recommended target were considered (LDLc of < 55 mg/dl and on treatment allowing at least 50% reduction from baseline LDLc levels, i.e., HIS and HIS + ezetimibe). Subjects with diabetes were more likely to achieve these targets (for 2019 targets: 7.4% and 10.7%, for patients with and without diabetes, respectively; relative risk: 1.44, 95% CI 1.28–1.62, p < 0.001). Moreover, as shown in Fig. 2 panel B, the overall proportion of patients with diabetes achieving < 70 mg/dl or < 55 mg/dl of LDLc was higher than those without diabetes (55% and 29% vs*.* 39% and 16%, respectively, all p < 0.001). Similar differences were confirmed in all different categories of treatments.

### Factor associated with absolute LDLc target attainment

**Fig. 3:**
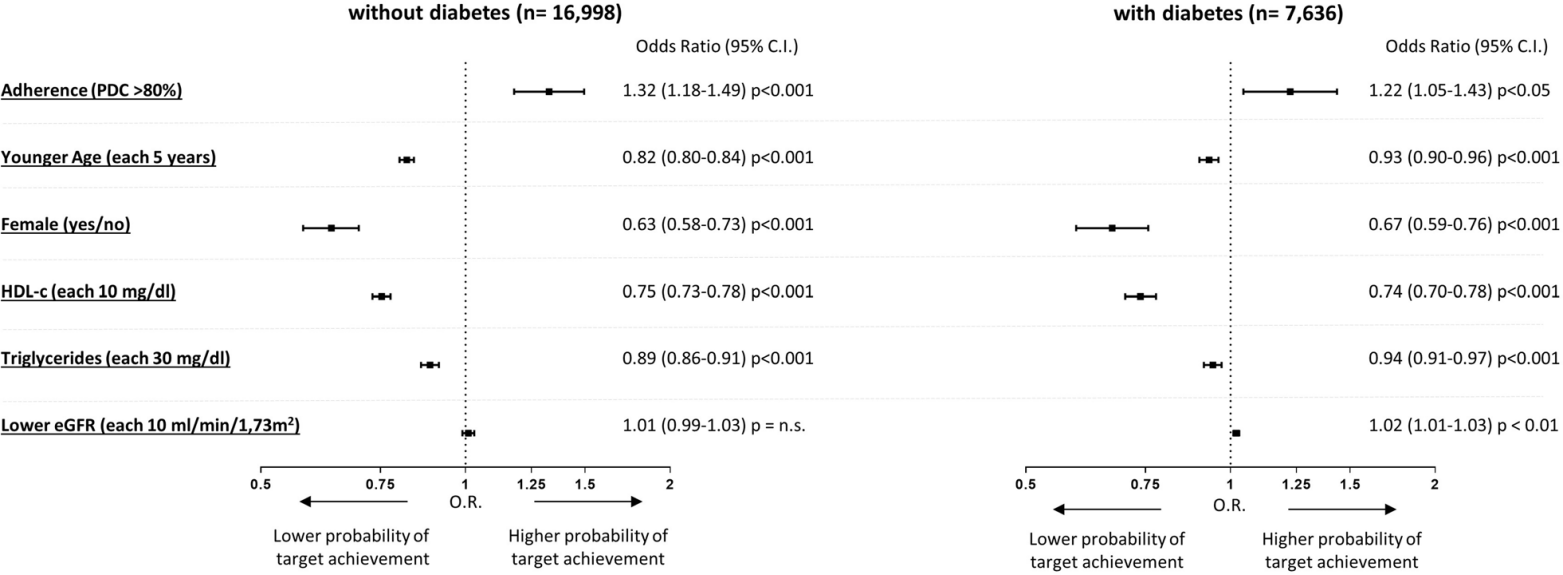
Factors associated with LDL-c levels < 70 mg/dl in patients without and with diabetes (model adjusted by age, sex, prior history of cardiovascular events, adherence, statin treatment intensity, HDL-c, triglycerides, eGFR)

Finally, after accounting for prior CVD events and different types of statin treatments, we evaluated which factors were associated with a higher or lower probability of having LDLc levels < 70 mg/dl. As shown in Fig. 3, both in patients with and without diabetes, subjects with higher adherence were more likely to achieve this target. Conversely, female subjects, younger subject, those with higher HDL-cholesterol or triglycerides were independently associated with a lower probability of being at LDLc < than 70 mg/dl. Among patients with diabetes, also lower eGFR was marginally but significantly associated with a higher probability of being at LDLc target.

## Discussion

The recent ESC/EASD Guidelines on diabetes, pre-diabetes, and cardiovascular diseases [7] but also the ESC/EAS Guidelines on the management of dyslipidaemias [22], endorse with a class I of recommendations, and a B level of evidence, that the target of LDLc, in patients with and without type 2 diabetes at very high CV risk, should be < 55 mg/dL (< 1.4 mmol/L) and at least 50% reduction from baseline [7]. Based on this premise, the key-findings of the present study were that among patients currently treated with statins: (1) Despite the overall high adherence to treatment in our population, only a small proportion of patients with a previous event achieve the target defined by these two criteria; (2) Few patients are treated with HIS, or with statin-ezetimibe combined treatment, and prescription of statins follow mainly a “treat to absolute” LDLc targets rather than achieving relative (%) reduction from pre-treatment LDLc levels; (3) Female sex, younger age, higher HDLc and triglycerides (all independently associated with lower probability of achieving LDLc targets both in patients with and without diabetes regardless of adherence, the intensity of treatments and presence of a prior event) are factors associated with unjustifiably delayed initiation of statin treatment only for higher LDLc levels; (4) Despite being still far from the recommended target, patients with diabetes have a significantly lower lipid values irrespective of statin treatments, prior event and its location, compared to patients without diabetes.

These findings allow conveying some novel and important messages to address and tackle the widely and continuously reported issue of low achievement of LDLc targets among patients with high or very high cardiovascular risk [10, 23, 24].

First, our study selected a population enriched of subjects with high adherence (by study design only subjects actively on statin treatments and collecting the medication from the pharmacy in the 6 months before lipid profile assessment were included). Indeed, the proportion of subjects with high adherence ranged between 78 and 100% and was progressively higher in those with a prior event or on higher intensity treatment. However, despite this, we found that only a small proportion achieved the 2016-targets and less than one out of ten subjects achieved the 2019-targets. Therefore, while adherence is intrinsically related to LDLc levels as reported in multiple studies [11, 25–27], it likely explains only a minor part of the low-achievement of LDLc targets in high-risk patients.

Second, as reported by others, our data suggest that the low-achievement of targets is strongly related, especially in patients with a prior event, by the underuse of HIS, either alone or in combination with Ezetimibe, both in patients with and without diabetes [14, 23, 28–30]. Our data, however, allow providing more details on reasons and factors requiring modification to implement the 2019-guidelines recommendation.

Notably, after the stratification of subjects according to the presence of diabetes and prior event, there were only small or minor differences in the observed mean LDLc levels across groups of subjects exposed to treatments having different lipid-lowering intensity (e.g., HIS vs. NHIS or HIS + ezetimibe vs. HIS alone). Such results might appeared counterintuitive if one consider the well-established efficacy of these treatments (consistently observed in randomized clinical trials regardless of most of the clinical characteristics of patients, including the presence of diabetes) [22, 31, 32]. However, our findings should be interpreted in the context of a cross-sectional observational study. Indeed the expected differences between HIS vs. NHIS or HIS + ezetimibe vs. HIS alone treatments are valid if the patients have similar before-treatment LDLc levels, that is hardly to imagine in real-world setting. In this context, one should consider an *indication bias* as a possible explanation, i.e. the possibility that the most intensive treatment (allowing ≥ 50% reduction) are more likely used among those with higher LDL-c at baseline (e.g., before treatment LDLc ≈ 140 leading to after treatment levels ≈ 70 mg/dl). Conversely, moderate intensity statin are more likely used in those subjects with lower LDL at baseline (e.g., before treatment LDLc ≈ 100 mmg/dl, with after treatment levels ≈ 70 mg/dl). Altogether, these results suggest that most of the patients are treated following a “treat to absolute LDLc targets” approach. In support of this, one can note that the mean LDLc levels of subjects with diabetes and a prior event are aligned around 70 mg/dl, and this is explainable with 2016-guidelines targets (valid at the time when these data were collected) requiring for those on secondary prevention an absolute LDLc < 70 mg/dl OR at least a 50% reduction from untreated levels. Conversely, recent guidelines beyond reducing the absolute LDLc targets clarified that both criteria (at least 50% reduction from untreated levels) must be achieved, regardless of baseline LDLc levels (for all patients with high or very high cardiovascular risk). A modification strongly supported by the established log-linear relationship between LDLc reduction and reduction of cardiovascular risk [1]. Therefore, our data highlights the importance of modifying physician prescription patterns towards a “treat to absolute targets AND a relative change in LDLc levels”.

In support of this recommendation, we have indeed recently reported how their implementation would have a dramatic impact on the cardiovascular burden in diabetes and would reduce, over 10 years, the number of CV events in patients with diabetes by one third [14].

Positive attainment should require a more robust adhesion to statin treatment and the optimization of their dosing also in patients on ezetimibe therapy. This is graphically depicted in Fig. 2, in which is clear that across different treatments, most of the patients with a previous event in both groups, without and with diabetes, were eligible for a maximum tolerated dose of statin plus Ezetimibe. Second, we show that only a small proportion of patients in the two groups were on high intensity statin plus Ezetimibe. Conversely, considering: (1) the current treatment used by patietns, (2) the observed LDL-c levels, and (3) the expected reduction in LDLc from intensification of lipid-lowering treatments, it is possible to estimate that in this large population based study ≈ 40% of patients (with and without diabetes) on secondary CVD prevention would be able to achieve the 55 mg/dl targets if treated with a combination of HIS + Ezetimibe. HIS alone would be sufficient to achieve the target only in 40% and 22% of patients with and without diabetes, respectively. Notably, the addition of PCSK9 inhibitors on top of HIS and ezetimibe, would be needed to achieve the targets in another 18% and 34% of patients with and without diabetes, respectively. Unfortunately, we were unable to quantify those who were statin intolerant: however, as many as 15% of individuals with a clinical indication for statin therapy are unable to take it because of some degree of intolerance [33]. Therefore, the proportion of subjects needing PCSK9-inhibitors treatment might be even larger after accounting for those patients being intolerant or with reduced tolerance to statins.

Third, for the same reasons described above (cross-sectional design of the study and expected independent response to statins regardless of patient characteristics) our data suggest that female sex, lower age, high HDLc levels, and more elevated triglycerides are factors likely associated with delayed initiation of statin treatments and only in case of higher baseline LDLc levels.

These analyses, given the cross-sectional and observational setting of real-world study, should be interpret as associations and cannot confirm any causal relationship. Moreover we were not able to consider other important cardiovascular risk factors such as smoking habits and blood pressure controls. However, the underuse of lipid-lowering treatment in female subjects has also been reported in other studies [14, 25], and here we confirmed that it is independent of adherence. One possible explanation might be the misperceived CVD risk of female subjects. While historically considered “protected” from CVD, this protection is progressively reduced or lost with aging and among those on secondary prevention and, particularly, among those with diabetes [34]. Therefore, increasing LDLc lowering treatment in female patients represents a pivotal factor in improving cardiovascular prevention strategies. Similarly, the known inverse association between HDLc and CVD risk might cause physicians to underestimated the importance of reducing LDLc in patients with relatively high HDLc. We also found that hypertriglyceridemia was inversely associated with the achievement of low LDLc levels. However, while selected patients might have cardiovascular benefit from TG-lowering approaches [35, 36], it must be stressed that LDLc targets are the first goal to be achieved in patients with mild-hypertriglyceridemia and only after that other treatments to reduce TG-rich lipoprotein should be initiated (e.g., fenofibrate or omega-3 eicosapentaenoic acid)[22]. Fourth, in patients with diabetes, we have observed significantly lower lipid values and a higher proportion of patients at target, both < 70 mg/dl and at < 55 mg/dl LDLc. This observation has already been reported in a real-world setting in Netherland [30], and from an extensive, national, administrative claims database in United States [37, 38]; the presence of diabetes has been reported as an independent predictor of achieving LDLc goal [39]. In the present study, we add some exciting observations, i.e., patients with diabetes had significantly lower LDLc and non-HDLc concentrations independently of the site of a prior event.

Furthermore, the level of LDLc was significantly lower among those with CVD compared to those with cerebrovascular disease. However, it must be acknowledged that the significance is present only in the groups of treatment with the highest numerosity. A lower achievement of LDLc in patients with stroke has been already observed in previous studies [40], and, when compared with patients with CAD, patients with the stroke earlier are less likely for patients to have LDLc < 100 or < 70 mg/dl. This observation has a particular relevance also in the light of the finding that intensive treatment reduces cerebrovascular events [41], and after the observation that in patients with an ischemic stroke or TIA, and LDLc level of < 70 mg/dl had a lower risk of subsequent cardiovascular events than those with an LDLc between 90 and 110 mg/dl [42].

The lower LDLc levels also observed in primary prevention suggest that patients with diabetes are treated earlier and at lower LDLc levels than patients with diabetes. The specific Italian diabetic clinic organization can partly justify this observation: attending the diabetic clinic is associated with a significant 30% decrease in mortality [43] and a significant 17% reduction in CV mortality. We have recently shown that for patients with type 2 diabetes attending a specialist outpatient clinic, intensive complication screening is followed by better long-term cardiovascular outcomes [8].

This study has limitations and strengths. First, its cross-sectional nature does not provide any information on each treatment’s role on incident events, nor we provide information on those with more than one event who would be eligible to even lower LDLc targets. Second, we could not evaluate the role of two important risk factors for CVD such as smoking habit and blood pressure control. While in patients in secondary prevention these would have not changed the LDL-cholesterol targets, this information would have improved risk stratification in patients on primary prevention (and therefore the identification of the appropriate LDL-cholesterol targets). Although this information are typically not available in large administrative databases, physician should nonetheless aim to a comprehensive control of patients’ cardiovascular risk and target them. Third, it should be outlined that, since our study is based on laboratory information collected from the Central Laboratory of the University Hospital of Padova, a possible hospital-bias might be present. Before generalizing these results to other setting, one should consider that usually patients with a higher clinical complexity are referred to third level hospital laboratory (as inpatient or as outpatient). Nonetheless it is remarkable that we were able to initially evaluate ≈ 26% of the entire population of the Padua province (934,332 subjects) having at least one determination of lipid profile in this laboratory. Fourth, we were not able to evaluate the actual adherence of intake of pills from patients given the large observational population-based study, however the PDC measure is considered as one of the most reliable method to measure adherence in chronic therapies and is expected to reflect the actual pill intake [44]. The strengths of this study are the high number of subjects included, the comparison in two different populations of two other lipid targets, the proportion of adherence evaluated form pharmacy-claim data, and detailed information on statin dosage.

In conclusion, the present study demonstrates that among patients currently on statin treatment and with relatively high adherence to treatment, the proportion of patients at LDLc target is very low, also among those on secondary prevention. We identified, both in patients with and without diabetes, the late initiation of high-intensity treatments, particularly among those with misperceived low risk (e.g., female subjects or those with high HDLc), as possible pivotal factors needing to be modified to improve CVD prevention.

## Supplementary Information

**Additional file 1: Figure S1.** Study flow-chart and design. **Figure S2.** LDL-cholesterol levels in patients with events according to event site and lipid-lowering treatments.

## Data Availability

The data underlying this article were provided by CliCon by permission. Data will be shared on request to the corresponding author with the permission of CliCon.

## Abbreviations

ATC: Anatomical-therapeutic chemical
CHD: Coronary heart disease
CVD: Cardiovascular disease
eGFR: Estimated glomerular filtration rate
HDL: High density lipoprotein
HIS: High-intensity-statin.
LDL: Low dense lipoprotein
NHIS: Non-high-intensity-statin.
PDC: Proportion of days covered
