# Supplementary material for: Improving statin treatment strategies to reduce LDL-cholesterol: factors associated with targets’ attainment in subjects with and without type 2 diabetes
Source: Cardiovasc Diabetol. 2021 Jul 16;20:144. doi: 10.1186/s12933-021-01338-y (PMC8283985; doi:10.1186/s12933-021-01338-y)
Supplement: Supplementary file 1 — Additional file 1: Figure S1. Study flow-chart and design. Figure S2. LDL-cholesterol levels in patients with events according to event site and lipid-lowering treatments. [file 12933_2021_1338_MOESM1_ESM.docx]

Additional file to Morieri et al. “**Improving statin treatment strategies to reduce LDL-cholesterol: factors associated with targets' attainment in subjects with and without type 2 diabetes.”**

**Additional file 1: Figure S1. study flow-chart and design**


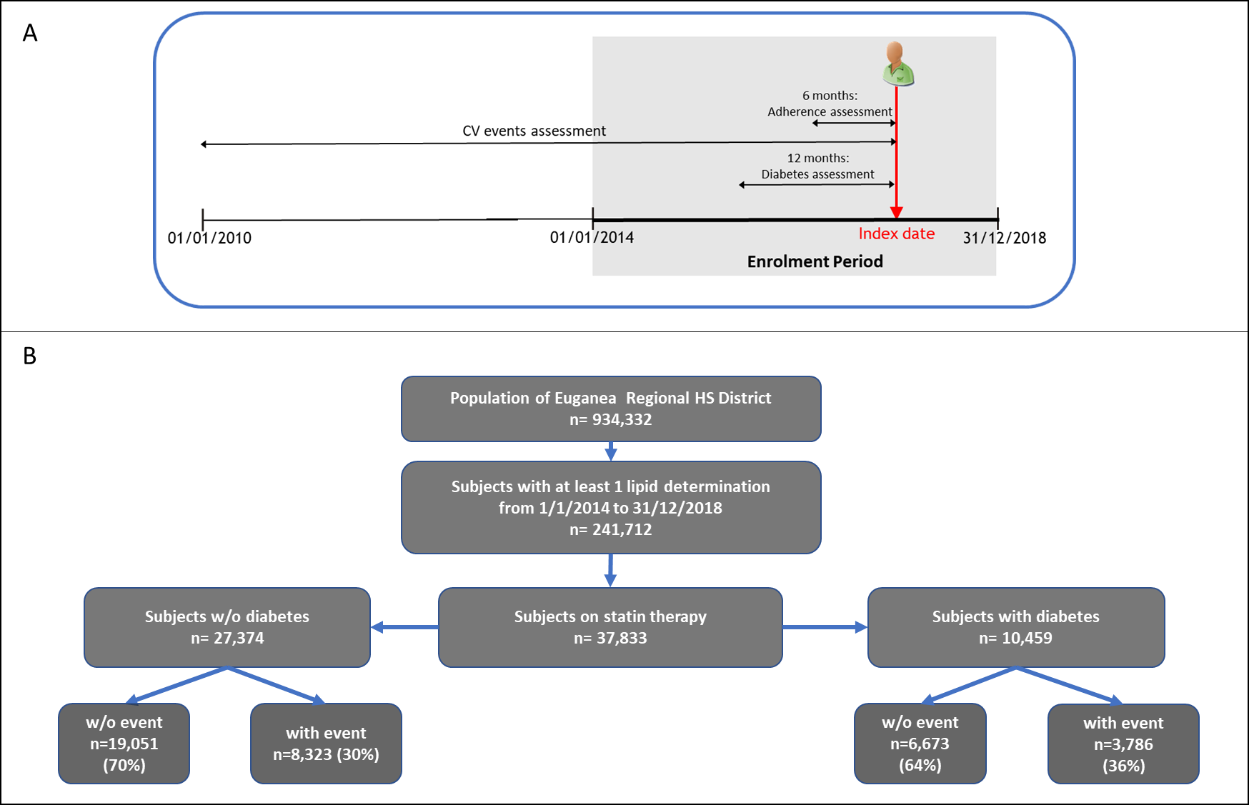


**Additional file 1: Figure S2. LDL-cholesterol levels in patients with events according to event site and lipid-lowering treatments**

**
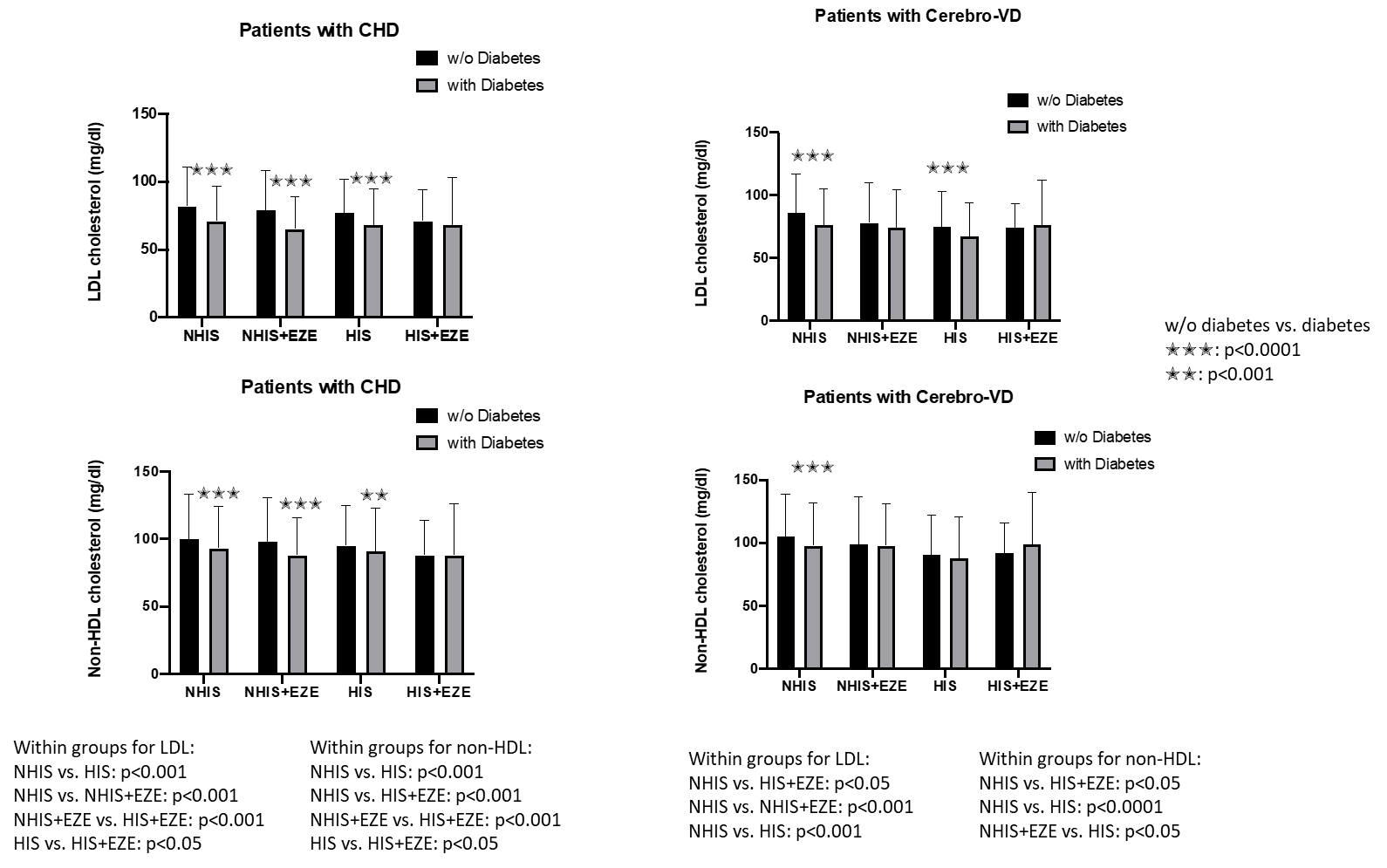
**
